# Noninvasive assessment of metabolic turnover during inflammation by *in vivo* deuterium magnetic resonance spectroscopy

**DOI:** 10.3389/fimmu.2023.1258027

**Published:** 2023-09-28

**Authors:** Vera Flocke, Sebastian Temme, Pascal Bouvain, Maria Grandoch, Ulrich Flögel

**Affiliations:** ^1^ Experimental Cardiovascular Imaging, Institute for Molecular Cardiology, Heinrich Heine University Düsseldorf, Düsseldorf, Germany; ^2^ Department of Anaesthesiology, University Hospital Düsseldorf, Düsseldorf, Germany; ^3^ University Hospital Düsseldorf, Cardiovascular Research Institute Düsseldorf (CARID), Düsseldorf, Germany; ^4^ Institute for Translational Pharmacology, Heinrich Heine University Düsseldorf, Düsseldorf, Germany

**Keywords:** inflammation, metabolism, neutrophils, glycolysis, deuterium, magnetic resonance spectroscopy and imaging

## Abstract

**Background:**

Inflammation and metabolism exhibit a complex interplay, where inflammation influences metabolic pathways, and in turn, metabolism shapes the quality of immune responses. Here, glucose turnover is of special interest, as proinflammatory immune cells mainly utilize glycolysis to meet their energy needs. Noninvasive approaches to monitor both processes would help elucidate this interwoven relationship to identify new therapeutic targets and diagnostic opportunities.

**Methods:**

For induction of defined inflammatory hotspots, LPS-doped Matrigel plugs were implanted into the neck of C57BL/6J mice. Subsequently, ^1^H/^19^F magnetic resonance imaging (MRI) was used to track the recruitment of ^19^F-loaded immune cells to the inflammatory focus and deuterium (^2^H) magnetic resonance spectroscopy (MRS) was used to monitor the metabolic fate of [6,6-^2^H_2_]glucose within the affected tissue. Histology and flow cytometry were used to validate the *in vivo* data.

**Results:**

After plug implantation and intravenous administration of the ^19^F-containing contrast agent, ^1^H/^19^F MRI confirmed the infiltration of ^19^F-labeled immune cells into LPS-doped plugs while no ^19^F signal was observed in PBS-containing control plugs. Identification of the inflammatory focus was followed by i.p. bolus injection of deuterated glucose and continuous ^2^H MRS. Inflammation-induced alterations in metabolic fluxes could be tracked with an excellent temporal resolution of 2 min up to approximately 60 min after injection and demonstrated a more anaerobic glucose utilization in the initial phase of immune cell recruitment.

**Conclusion:**

^1^H/^2^H/^19^F MRI/MRS was successfully employed for noninvasive monitoring of metabolic alterations in an inflammatory environment, paving the way for simultaneous *in vivo* registration of immunometabolic data in basic research and patients.

## Introduction

Inflammation and metabolism are closely intertwined processes that influence each other in multiple ways (1, 2). During an inflammatory response, the body requires additional energy to support the activation and proliferation of immune cells involved in the immune response and successful healing. Metabolic pathways are redirected to generate the necessary energy and substrates for these processes (3), while immune cells, especially macrophages and T cells, undergo metabolic reprogramming during inflammation (4). On the other hand, inflammatory cytokines, such as interleukin-6 (IL-6) and tumor necrosis factor-alpha (TNF-α), can affect insulin sensitivity, glucose, and lipid metabolism (5), potentially leading to insulin resistance. In line with this, chronic low-grade inflammation is linked to various metabolic disorders, including obesity, type 2 diabetes, and cardiovascular diseases (6, 7).

In order to harness this interplay for clinical decision-making, as well as to further delineate the mechanisms on how inflammation and metabolism interact, noninvasive approaches that monitor both processes *in vivo* would be highly desirable. In this context, the metabolism of glucose is especially of major interest, since, in particular, in the initial inflammatory phase, infiltrating immune cells mainly rely on glycolysis to cover their energy demand (8, 9). Positron emission tomography (PET) using [2-^18^F]fluorodeoxyglucose (FDG) is an established and sensitive tool for glucose imaging (10), which has also been applied for visualization of inflammation via the enhanced glucose uptake of the affected tissue (11). However, it has several limitations since it requires radioactive tracers and is restricted to monitor glucose uptake, whereas further metabolic turnover of the ingested glucose remains unclear (12). Furthermore, an increased glucose uptake is not specific for inflamed tissue and occurs also, e.g., in cancer [known as the Warburg effect (13)] and thus is a rather indirect readout for inflammation.

In the present study, the power of multinuclear magnetic resonance (MR) techniques was used to specifically resolve inflammatory and metabolic processes in parallel but at different levels: The ^19^F nucleus was employed to track immune cell trafficking, ^2^H (deuterium) for monitoring metabolic turnover, and conventional ^1^H MR imaging (MRI) for anatomical assessment of the inflamed tissue. ^19^F MRI emerged over the last decade as a background-free approach for inflammation imaging (14, 15), while ^2^H MR spectroscopy (MRS) is a novel, noninvasive method for tracking the pathway of deuterated substrates, which has yet be mainly used for analysis of cerebral or cancer metabolism (16–18). Here, these approaches were combined for the first time to monitor metabolic changes via ^2^H MRS in inflamed tissue identified by ^1^H/^19^F MRI. To this end, an easy and reproducible murine inflammation model was applied, making use of an implanted Matrigel plug doped with LPS (19) for an efficient recruitment of circulating immune cells into this experimental inflammatory focus.

## Methods

### Animal experiments

Eight- to 12-week-old male C57BL/6J mice (Janvier) were housed at the central animal facility of the Heinrich Heine University (Düsseldorf, Germany). All animal studies were approved by the “Landesamt für Natur, Umwelt und Verbraucherschutz Nordrhein-Westfalen” and were performed in accordance with the national guidelines on animal care (file references 81-02.04.2018.A007 and 81-02.04.2023.A050). The mice were fed with a standard chow diet and received tap water *ad libitum*.

### Inflammation model (Matrigel/LPS)

To implant the Matrigel plug, mice were anesthetized with isoflurane and placed on a 37°C warming plate. Fifty microliters of a fluid Matrigel solution mixed with LPS (1 µg/µL; BD Biosciences) or PBS as control were injected s.c. into the neck, which turned into a solid gel at body temperature forming a jellylike plug stable over a period of several weeks (19).

### Magnetic resonance imaging and spectroscopy

#### General

Data were recorded at vertical Bruker AVANCE^III^ and AVANCE NEO 9.4T wide bore NMR spectrometers driven by ParaVision 5.1 and 360v3.2, respectively, and operating at a frequency of 400.21 MHz for ^1^H, 376.54 MHz for ^19^F, and 61.43 MHz for ^2^H measurements. Images were acquired using Bruker microimaging units Micro 2.5 with actively shielded gradient sets (1.5 T/m) and resonators/coils depending on the application (all Bruker, see below). Mice were anesthetized with 1.5% isoflurane and kept at 37°C.

#### ^1^H/^19^F MRI

Data were acquired using a 25-mm resonator tuneable to both ^1^H and ^19^F. For visualization of inflammatory processes, mice received an intravenous bolus injection of a 10% perfluoro-15-crown-5 ether emulsion (PFC, 3 mM/kg BW) 24 h prior to MRI to ensure appropriate PFC-loading of circulating immune cells (15). After acquisition of ^1^H datasets, the resonator was tuned to ^19^F, and morphologically matching ^19^F images were recorded. For superimposing the images of both nuclei, the “hot iron” color look-up table provided by ParaVision was applied to ^19^F images. ^1^H MR reference images were recorded by a multislice rapid acquisition with relaxation enhancement (RARE) sequence: RARE factor 16, field of view (FOV) 2.56 × 2.56 mm^2^, matrix 256×256, slice thickness (ST) 0.5–1 mm, 1–4 averages, and acquisition time (TAcq) 0.5–6 min. Corresponding ^19^F images were recorded from the same FOV using a RARE sequence with the following parameters: RARE factor 32, matrix 64 × 64, ST 2 mm, 256 averages, and TAcq 21 min. For a more detailed description of the ^19^F MRI approach, acquisition parameters, and quantification procedures, please refer to Refs (15, 20).

#### ^1^H MRI combined with ²H MRS

In separate experiments, a 12 × 8 mm^2^ transmit/receive ^2^H surface coil inserted into a 30-mm ^1^H saw resonator was utilized for metabolic measurements. The mice were placed with their neck (containing the Matrigel plug) on the surface coil, and for application of deuterated glucose during the MR session, a Vasofix Safety IV Catheter (Braun Melsungen AG) was inserted into the peritoneal cavity. After insertion in the magnet, the correct positioning was verified by ^1^H MRI. Subsequently, fieldmap-based shimming (MAPSHIM) followed by manual adjustment was carried out to optimize the field homogeneity in the region of interest. Thereafter, ^2^H MR spectra were recorded over the entire Matrigel region for determination of glucose metabolism [rectangular pulse, 60° flip angle; repetition time (TR), 350 ms; spectral width, 15 ppm; data size, 300 points; averages, 300; TAcq, 2 min]. After acquisition of baseline spectra, mice received an i.p. bolus injection of 2 mg/g [6,6-^2^H_2_]glucose per body weight followed by continuous monitoring of deuterated metabolites over 60 min. Spectra were processed and deconvoluted with TopSpin (Bruker). Exponential weighting resulting in a 10-Hz line broadening was applied and chemical shifts were referenced to the resonance frequency of water at 4.7 ppm. Signal intensities were obtained by integration and normalized to their background values under baseline conditions, or for calculation of metabolic fluxes, absolute intensities were used.

### Flow cytometry

After termination of all MRI/MRS measurements, mice were sacrificed without disrupting the Matrigel plug located in the neck. Matrigel plugs were carefully excised, digested with Collagenase D (0.075 U/mL) and DNase I (200 U/mL; both Roche Applied Sciences), and prepared for flow cytometry as previously described (19, 21, 22). In brief, cells were resuspended in ice-cold MACS buffer (phosphate-buffered saline, 2 mM EDTA, and 0.5% bovine serum albumin) and stained with antibodies against the following molecules to identify T cells, B cells, monocytes, or macrophages: anti-CD45(PE), anti-CD3(FITC), anti-B220(APC), anti-CD11b(APC), anti-Gr1(PE.Cy7), anti-Ly6c(APC.Cy7), anti-CD11c(FITC), anti-MHCII(PE), and anti-F4/80(PerCP5.5). All antibodies were purchased from Miltenyi Biotec (Bergisch Gladbach, Germany) or BioLegend (Amsterdam, Netherlands) and diluted (1:200–400) in ice-cold MACS buffer. Cells were stained for 30 min at 4°C, washed three times with cold MACS buffer, and finally resuspended in 250 µL of MACS buffer. Dead cells were labeled with DAPI (4′,6-diamidino-2-phenylindole; 1 µg/mL) and excluded from the analysis. FACS datasets were acquired on a CantoII (BD Biosciences) and analyzed using FACSDiva (BD Biosciences) or FlowJo^TM^ (v10.8; Ashland, OR, USA). The following expression patterns were used for identification of the immune cell subtypes: T cells: CD45^+^ and CD3^+^; B cells: CD45^+^ and B220^+^; neutrophil granulocytes: CD11b^+^, Gr1^hi^, and Ly6c^+^; inflammatory monocytes: CD11b^+^, Gr1^+^, and Ly6c^hi^; macrophages: CD11b^+^, Gr1^low/neg^, Ly6c^low/neg^, CD11c^+^, MHCII^+^, and F4/80^+^.

### Immunofluorescence microscopy

Excised Matrigel plugs were embedded in Tissue-Tek (Weckert Labortechnik, Kitzingen, Germany) and frozen at −20°C. Sections of 8–10 µm were cut and fixed as previously described (15, 19). Matrigel samples were fixed for 10 min in Zamboni’s fixative, washed with PBS, and blocked (10% goat serum in PBS containing 0.1% saponin) for 10 min. Subsequently, Matrigel sections were stained with anti-CD11b mAb (1:100; Serotec; Düsseldorf, Germany) overnight at 4°C. After washing with PBS, samples were incubated with anti-rat-IgG-phycoerythrin (PE)-coupled secondary mAb (1:1,000 in blocking buffer; ThermoFisher) for 30 min at 4°C and washed again three times with PBS. Finally, sections were embedded in ProLong Gold antifade reagent (with DAPI; Invitrogen). Slices were analyzed using an Olympus BX61 fluorescence microscope equipped with a 12-bit CCD monochrome (F-View II) driven by CellSense Dimension software. Images were analyzed and processed with Fiji (23).

### Statistics

Statistical analysis was performed using GraphPad 9.5. Normal distribution was tested using Shapiro–Wilk test. For comparison of treatment groups, a two-way ANOVA or an unpaired-samples Students *t*-test was used. A *p*-value < 0.05 was considered statistically significant.

## Results

**Figure 1:**
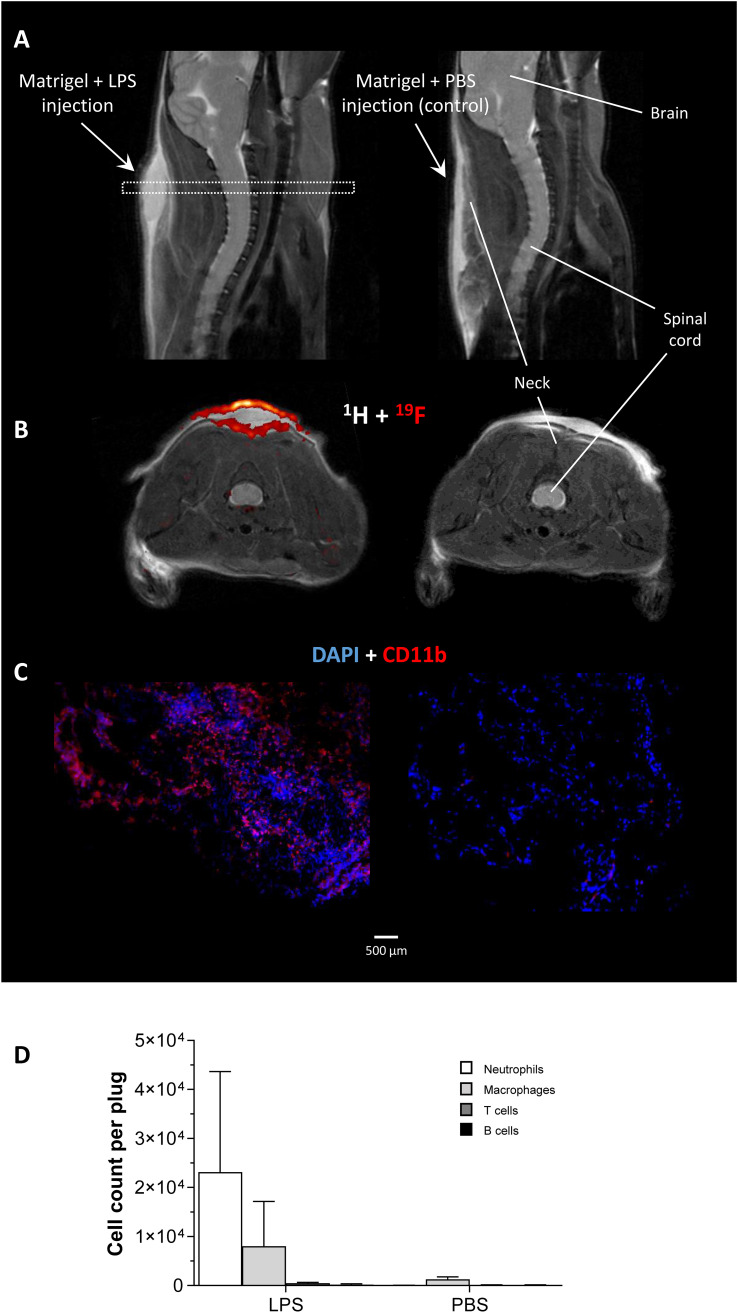
*In vivo* and *ex vivo* validation of the experimental inflammatory focus. **(A)** Sagittal T2-weighted ^1^H MR images visualizing the injected Matrigel doped with 1 µg/µL LPS/PBS in the neck of healthy C57BL/6J mice as bright structure. The dotted rectangle indicates the slice localization in **(B)**. **(B)** Axial ^1^H/^19^F MRI on day 1 after Matrigel implantation revealed only in LPS-doped plugs an infiltration of ^19^F-loaded immune cells. **(C)** Immunohistochemistry of excised plugs confirmed the *in vivo* findings in **(B)**. **(D)** Flow cytometric analysis of immune cells isolated from LPS/PBS-doped Matrigel plugs indicating neutrophils as the predominant cell population recruited to the inflammatory focus at that time point.

For induction of defined inflammatory conditions, we made use of a previously described murine model with neck implantation of an LPS-doped Matrigel plug, while using PBS-doped plugs as respective negative control (19). The successful formation of an inflammatory hotspot within the neck of mice was verified by ^1^H/^19^F MR inflammation imaging (14) 1 day after implantation of the doped Matrigel plugs. To this end, PFCs that are preferentially phagocytosed by circulating neutrophils/monocytes were injected intravenously for visualization of the infiltrating immune cells. **Figure 1A** shows the anatomical location of the Matrigel plug that clearly emerged as a bright oval structure in T2-weighted ^1^H MRI of the neck region. Merging of subsequently acquired ^1^H and ^19^F images (**Figure 1B**) demonstrated the presence of ^19^F-loaded immune cells predominantly in the border region of the LPS-doped Matrigel plug, while no ^19^F signal was found in PBS-doped control plugs. These *in vivo* findings were underpinned by both histology and flow cytometry corroborating the marginal location of immune cells in the plug (**Figure 1C**) and revealing neutrophils as the predominant cell population infiltrating the plug at this early time point after implantation (**Figure 1D**).

**Figure 2:**
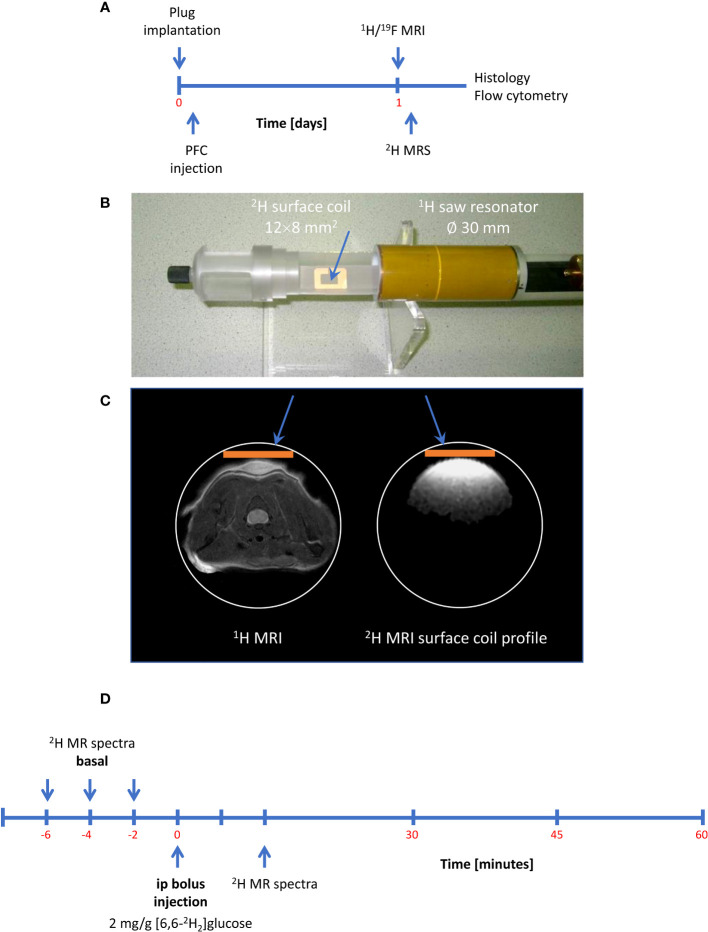
Setup and workflow for ^2^H MRS. **(A)** Timeline with plug implantation, inflammation imaging, metabolic measurements, and histology/flow cytometry. **(B)** ^2^H MRS was carried out with a ^2^H surface coil inserted into a ^1^H volume resonator. **(C)** Anatomic localization of the plug with the volume resonator (left) and excitation profile of the ^2^H surface coil (orange bar) acquired from a D_2_O phantom (right) demonstrating that the penetration depth of the surface coil is restricted to the dimensions of the Matrigel plug and the surrounding scapular muscles. **(D)** Workflow for ^2^H MRS: After acquisition of baseline spectra, mice received an intraperitoneal bolus of 2 mg/g [6,6-^2^H_2_]glucose per body weight followed by continuous acquisition of ^2^H MR spectra.

**Figure 3:**
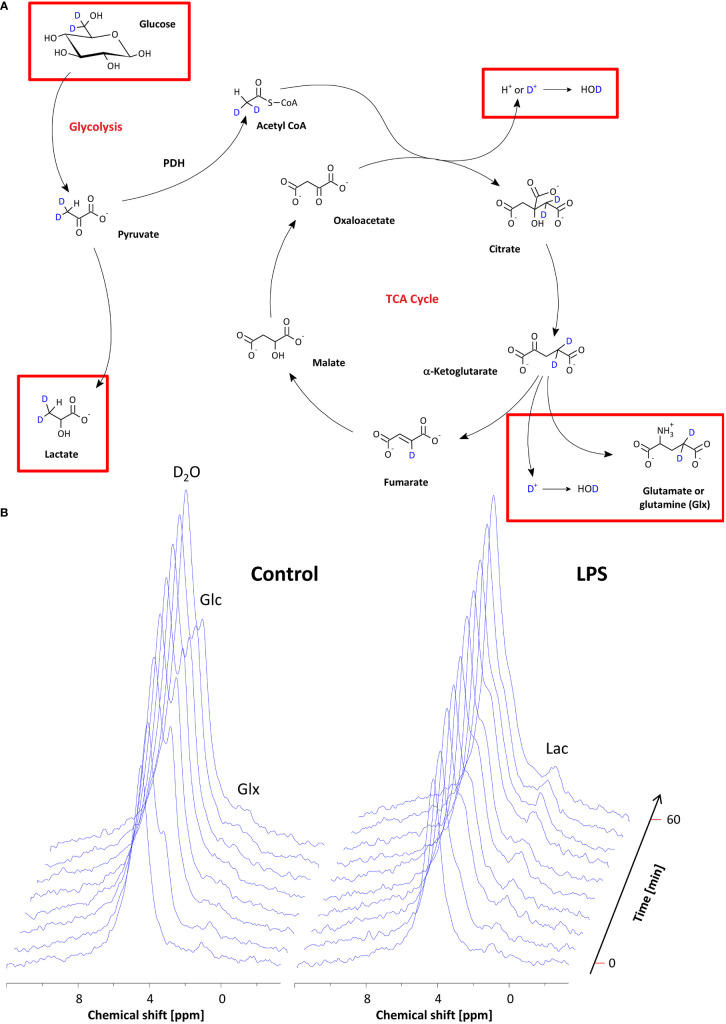
Monitoring of the metabolic fate of [6,6-^2^H_2_]*g*lucose by ^2^H MRS. **(A)** Scheme of the metabolic pathway for [6,6-^2^H_2_]glucose through glycolysis and the TCA cycle. The red rectangles indicate the metabolites that are within the detection range of ^2^H MRS. **(B)** Stack plot of ^2^H MR spectra over the entire observation period for mice with PBS-doped (left) and LPS-doped (right) Matrigels, respectively. See the text for detailed explanations.

After confirming the presence of inflammatory foci (**Figure 2A**), mice were placed with their neck on a 12 × 8 mm^2^ transmit/receive ^2^H surface coil inserted into a 30-mm ^1^H saw resonator (**Figure 2B**). This setup allowed the unambiguous localization of the Matrigel plug by ^1^H MRI (**Figure 2C** left) and the sensitive acquisition of ^2^H MR spectra with the surface coil, whose penetration depth and excitation profile essentially encompassed the Matrigel plug and surrounding scapular muscles (**Figure 2C** right). After acquisition of baseline spectra, mice received an i.p. bolus injection of 2 mg/g [6,6-^2^H_2_]glucose per body weight followed by continuous monitoring of ^2^H MR spectra over 60 min (**Figure 2D**). **Figure 3A** illustrates the route of the injected ^2^H label (blue) through glycolysis and TCA cycle with the ^2^H MRS-detectable metabolites (in the millimolar range) highlighted in red, i.e., glucose, lactate, and glutamate/glutamine (Glx)—the latter cannot be spectrally resolved *in vivo* because of the broad linewidth of ^2^H MR spectra. Importantly, during formation of the enol form of acetyl-CoA prior to entry into the TCA cycle, one of the two deuteria can be split off (with a probability of 2/3)—this is eventually found in water and yields only a monolabeled citrate and Glx (not shown in the schematic).

**Figure 4:**
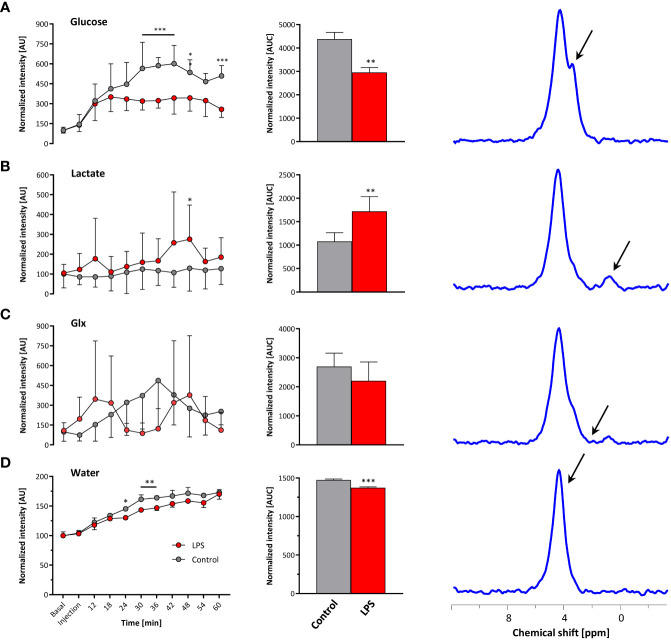
Quantification of ^2^H MR spectra over time. All measurements were normalized to spectra acquired under baseline conditions accounting for the natural background of ^2^H (0.01%). Gray, control mice with Matrigel/PBS; red, mice with Matrigel/LPS; left, time course over 60 min; middle, area under the curve (AUC); right, representative ^2^H MR spectrum with the respective metabolite for **(A)** glucose, **(B)** lactate, **(C)** Glx, and **(D)** water. Data are presented as means ± SD; *p < 0.05, **p < 0.01, ***p < 0.001; *n* = 9–12; two-way ANOVA for the time courses and unpaired Student’s *t*-test for the respective AUC.

Baseline spectra showed a prominent signal at 4.7 ppm (**Figure 3B** bottom lanes) caused by the natural abundant ^2^H in water. Injection of [6,6-^2^H_2_] glucose gave rise to a fast increase of the corresponding ^2^H signal at 3.8 ppm and the subsequent appearance of the downstream metabolites Glx (i.e., glutamine + glutamate) and lactate at 2.4 and 1.3 ppm, respectively, accompanied by a continuously increasing water signal. The temporal development of ^2^H MR spectra over the entire observation period of 60 min is illustrated in **Figure 3B** with typical examples for mice with Matrigel plugs doped with LPS (right) and PBS as control (left). As can be recognized, the glucose (Glc) signal declined much quicker in the presence of LPS while concomitantly lactate levels were clearly elevated as compared to control conditions. Quantification of spectra for *n* = 9–12 independent experiments (**Figures 4A–D**) confirmed these findings and calculation of Lac/Glc ratios over the entire observation period after application of the labeled glucose (LPS 1.18 ± 0.08 vs. PBS 0.90 ± 0.12; *p* < 0.05) further supported the notion of an enhanced lactate formation at the expense of increased glucose consumption under inflammatory conditions. Of note, this was also accompanied by a significantly lower incorporation of ^2^H label into water (**Figure 4D**).

Taking the naturally occurring ^2^H signal from water (**Figure 3B** bottom lanes) as an internal reference, absolute turnover rates can also be calculated: Assuming a content of 110 M hydrogen in H_2_O, an approximately 75% tissue water content (24), and a ^2^H natural abundance of 0.0115% in water (25, 26), the detected baseline signal reflects an amount of ~9.5 mM deuterons (27). Considering now the total increase of the water signal (**Figure 4D**, middle) over the observation period of 60 min, the deuterium turnover from [6,6-^2^H_2_]glucose to water is calculated to be 0.81 ± 0.07 mM/min under control conditions, while it was significantly diminished to 0.64 ± 0.04 mM/min under LPS exposure (*p* < 0.05, *n* = 9–12).

## Discussion

In the present work, ^2^H MRS was applied in combination with ^19^F MR inflammation imaging *in vivo* to monitor the metabolic fate of deuterated glucose within neutrophil-infiltrated inflammatory hotspots noninvasively and in real time. The findings of this study demonstrate that ^2^H MRS can indeed be successfully used to reveal metabolic signatures in inflammatory environments, specifically identified by hot spot ^19^F MRI together with anatomical ^1^H MRI.

The obtained results clearly point to an enhanced utilization of glycolysis for energy production in the region of the inflammatory hotspot. Increased lactate levels and enhanced glucose turnover are classical indicators for a predominant anaerobic metabolism, which is further supported by the lower flux of the ^2^H label to water. In the long term, all deuterons of [6,6-^2^H_2_]glucose will end up in the body water pool, but in the initial phase after bolus injection, the highest probability for the ^2^H label being transferred to water is given when it enters the aerobic pathway: if the label runs through the TCA cycle, it can be cleaved off either during formation of citrate or when it does not leave the cycle via α-ketoglutarate, but instead passes into fumarate (**Figure 3A**). Thus, the diminished ^2^H incorporation into water is overtly indicative for a lower utilization of glucose via the TCA cycle. In contrast, under control conditions, glucose turnover within the field of view covered by the surface coil (**Figure 2C**) is mainly determined by aerobic metabolism of the surrounding scapular muscles.

While this finding is not surprising *per se*, it nonetheless highlights the potential of multinuclear MR approaches to discriminate complex immunometabolic processes at separate levels in parallel (here, ^1^H, ^2^H, and ^19^F for anatomy, metabolism, and immune cells, respectively). Of course, compared to FDG-PET (10), the present approach is restricted to metabolites that are present in the millimolar range, but it lacks any harmful radiation and also allows monitoring of downstream metabolites as well as absolute turnover rates. Alternative MR approaches relying on the ^13^C nucleus offer better spectral resolution than ^2^H, but either require substantial longer acquisition times even without volume selection (normal ^13^C) or are limited to non-ideal substrates (e.g., pyruvate) as well as very short temporal windows of observation (hyperpolarized ^13^C; approximately 1 min). Nevertheless, owing to the dramatically enhanced sensitivity of the latter method, it provides superior spatial resolution within this time frame (28). On the other hand, a variety of deuterated substrates are readily available commercially for ^2^H MRS and applications for deuterated acetate, choline, fumarate, and β-hydroxybutyrate have already been described (29, 30), but the field is definitely still in its infancy here. Of note, ongoing advances in technology, such as denoising, undersampling, or indirect readout [as used in (28, 30, 31)] will further improve the sensitivity for detection of deuterated metabolites.

In the present study, a ^2^H surface coil was utilized together with a ^1^H volume resonator—while this setup provided excellent coverage and superior sensitivity for the near-surface inflammation model used, it is not suitable for metabolic analysis of deeper organs or tissue. However, this approach can be further developed by designing triple-tuned volume resonators to allow simultaneous acquisition (32) of ^1^H/^2^H/^19^F MR images for true temporal co-registration of inflammatory/metabolic processes without penetration limits—not only for basic research to analyze their interwoven relationships *in vivo*, but also in the long run also for clinical decision-making in immunologic and metabolic disease states, as the feasibility of both ^19^F inflammation and deuterium metabolic imaging in the clinical setting has already been demonstrated (16, 33–36).

## Conclusions and perspectives

In summary, the results of this study demonstrate that ^2^H MRS can be successfully used to reveal real-time metabolic signatures in inflammatory environments—specifically identified by ^1^H/^19^F MRI. In contrast to the current gold standard FDG-PET that detects glucose uptake only, the present approach discriminates inflammation and metabolism at different levels in parallel and enables the much more meaningful assessment of metabolic turnover through glycolysis and TCA cycle with analysis of downstream metabolites.

In the future, this approach can be employed to noninvasively and longitudinally characterize in depth the immunometabolic interplay in diseases, such as diabetes or obesity, but also its impact on autoimmunity, (innate) immune memory, tumor microenvironment, and hematopoiesis for identification of new therapeutic targets. As the MR techniques used in this study could be readily transferred to human scanners, they could also serve to assess the degree of immunometabolic responses in patients to tailor individual treatment regimens.

## Data availability statement

The original contributions presented in the study are included in the article/supplementary material. Further inquiries can be directed to the corresponding author.

## Ethics statement

The animal study was approved by Landesamt für Natur, Umwelt und Verbraucherschutz Nordrhein-Westfalen. The study was conducted in accordance with the local legislation and institutional requirements.

## Author contributions

VF: Data curation, Formal Analysis, Investigation, Validation, Visualization, Writing – original draft. ST: Data curation, Formal Analysis, Investigation, Validation, Visualization, Writing – review & editing. PB: Methodology, Resources, Writing – review & editing. MG: Conceptualization, Project administration, Supervision, Writing – review & editing. UF: Conceptualization, Funding acquisition, Project administration, Supervision, Validation, Visualization, Writing – review & editing.
